# Comparison of Serum Magnesium Levels in Overweight and Obese Children and Normal Weight Children

**DOI:** 10.7759/cureus.1607

**Published:** 2017-08-24

**Authors:** Syed Awais ul Hassan, Iftikhar Ahmed, Adeel Nasrullah, Shujaul Haq, Haider Ghazanfar, Abu Baker Sheikh, Rizwan Zafar, Ghazan Askar, Zamara Hamid, Arshad Khushdil, Amna Khan

**Affiliations:** 1 Department of Peadiatrics, Military Hospital Jhelum; 2 Department of Pediatric Surgery, Military Hospital Rawalpindi, Pakistan; 3 Department of Internal Medicine, Shifa International Hospital; 4 Internal Medicine, Newark Beth Israel Medical Center; 5 Department of Medicine, Shifa International Hospital; 6 Pediatrics department, Combined military hospital, skardu; 7 Pediatrics, Shifa International Hospital

**Keywords:** magnesium, obesity, overweight, metabolic syndrome, insulin resistance, diabetes

## Abstract

Purpose

Abnormalities in serum magnesium levels have been seen in obesity and its related diseases. Our aim is to determine the mean magnesium levels in overweight and obese children as compared to the levels in normal weight controls to study its relationship with obesity and overweight. The study was done at a tertiary care hospital.

Methods

A case-control study was conducted at the Department of Pediatrics, Combined Military Hospital, Peshawar, over a 12-month period from August 7, 2015 to August 6, 2016. A total of 140 children between 2-14 years of age were included in the study. They were divided into two equal groups of 70 children each. Both of the groups were matched according to their age and sex. Children with a body mass index (BMI) greater than or equal to 85th centile and 95th centile were placed in the overweight and obese category, respectively, and termed as cases while the other 70 children with a BMI greater than or equal to 5th centile but less than 85th centile were categorized as the normal weight group and termed controls. The serum magnesium levels of both case and control groups were calculated.

Results

The serum magnesium levels were significantly lower in the overweight and obese group (2.08 ± 0.211 mg/dl) as compared to the normal weight group (2.55 ± 0.155 mg/dl, p<0.001). A significantly strong inverse relationship was seen between serum magnesium levels and body mass index.

Conclusion

Mean serum magnesium levels in overweight and obese children are lower than those in normal weight children. Further studies are required to see the effect of supplementation of diet with this essential micronutrient on the weight of children.

## Introduction

Childhood obesity is one of the most alarming challenges developing in today's world. The problem is universal and is steadily affecting many low and middle-income countries, particularly in urban settings [1]. In urban centers, we can see a rise in sedentary lifestyles and unhealthy eating habits at an earlier age. Childhood obesity has led to a great number of obesity-related complications and the prevalence has increased at an alarming rate. Globally, in 2010 the number of overweight children under the age of five was estimated to be over 42 million. Close to 35 million of these were living in developing countries.

The current epidemic of childhood obesity has been associated with an alarming rise in the prevalence of pediatric type 2 diabetes. Hyperinsulinemia and insulin resistance (IR) are the precursors of type 2 diabetes [2]. Furthermore, insulin resistance is also the hallmark of the metabolic syndrome that is associated with visceral obesity, hypertriglyceridemia, low high-density lipoprotein (HDL), hyperglycemia, and hypertension. Overweight and obese children are more likely to remain obese into adulthood and are prone to develop diseases like diabetes, dyslipidemias, hypertension, and cardiovascular diseases at a younger age [3-4]. Low magnesium levels have also been seen to have an inverse relationship with steady state plasma glucose. Low magnesium levels also lead to insulin resistance [5].

Micronutrients and macronutrients have been implicated as an important factor in regulating various metabolic processes and thus playing a role in the etiology of obesity. Many studies are being conducted worldwide that clearly show a direct link between obesity and micronutrient deficiencies [6-7]. Deficiencies of various micronutrients, such as fat-soluble vitamins, B complex, vitamin C and ions such as calcium and magnesium, can be associated with increased body mass index (BMI). These micronutrients have an essential role in the metabolism of a variety of nutrients and also have a key role in regulating hunger and the hormones that control it. Our aim is to determine the mean magnesium level in overweight and obese children as compared to that in normal weight controls to study its relationship with obesity. The study was done at a tertiary care hospital.

## Materials and methods

This case-control study was carried out at the Pediatrics Department, Combined Military Hospital, Peshawar, from August 7, 2015 to August 6, 2016. A total of 140 children between 2-14 years of age were included in the study. They were divided into two equal groups of 70 children each. Both the groups were matched according to their age and sex. Children with a body mass index (BMI) greater than or equal to 85th centile and 95th centile were placed in the overweight and obese category, respectively, and termed as cases while the other 70 children with a BMI greater than or equal to 5th centile but less than 85th centile (as per the World Health Organization (WHO) standards) were categorized as the normal weight group and termed controls. Children with genetic, endocrine, or syndromic causes of obesity; those with diabetes mellitus; medical conditions predisposing to hypomagnesemia (gastroenteritis, chronic kidney disease, and chronic liver disease); or on medications predisposing to hypomagnesemia (for example diuretics, amphotericin) were excluded. Permission from the hospital administration and ethical committee was sought. Informed written consent was obtained from the parents of children. Demographic data was collected regarding age and gender. A detailed history was taken and an examination was performed. Investigations including blood complete picture, blood sugar random, chest X-ray, renal function tests, and liver function tests were done in order to rule out other diseases. Serum magnesium levels of both normal weight and overweight children were measured early morning on an empty stomach using Hitachi -192 serum analyzer (Hitachi, Japan). The normal serum magnesium levels ranged from 1.5 to 2.5 mg/dl.

The data was analyzed using the Statistical Package for the Social Sciences (SPSS), version 21.0 (IBM, Armonk, NY). Descriptive statistics were used to analyze and describe the data. Frequencies and percentages were calculated for qualitative variables like gender. Mean and standard deviation (SD) were calculated for quantitative variables like age, weight, BMI, and serum magnesium levels. The independent sample t-test was used to determine the difference between the two groups and a p-value < 0.05 was considered significant.

## Results

**Figure 1:**
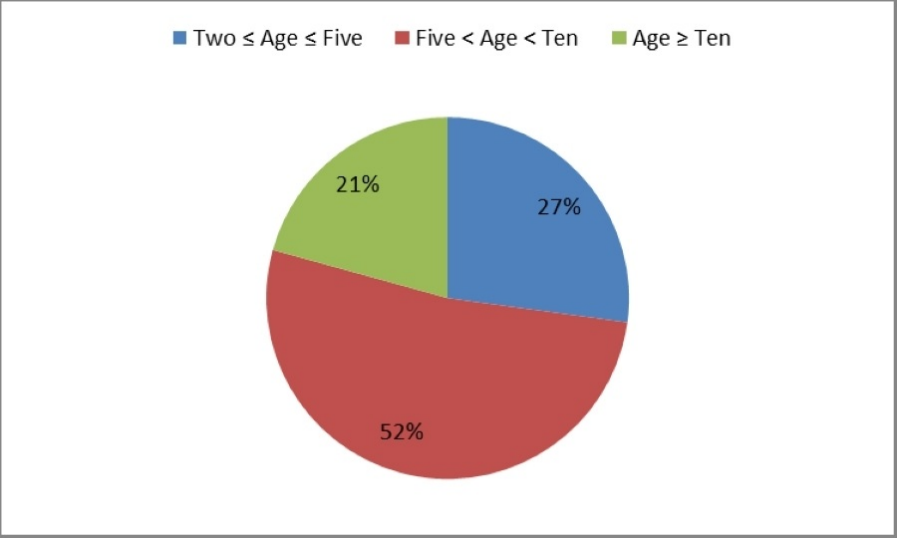
Age Distribution of the Study Population (Age in Years)

There were 51.4% (n=72) males and 48.6% (n=68) females. The age distribution was thus: 27% (n=38) children from two years to five years of age, 52% (n=73) between five and ten years, and only 21% (n=29) greater than or equal to 10 years of age. This has been shown in Figure 1.

The mean BMI of the study population was 21.84 ± 5.79, as shown in Table 1. The difference in BMI between the two groups was found to be statistically significant (p-value <0.001).

**Table 1:** BMI Distribution of the Study Population BMI: Body Mass Index

Variable	Overweight and Obese (n = 70)	Normal Weight (n = 70)	Total (n = 140)	p - value
BMI	27.11±1.64	16.57±2.93	21.84±5.79	<0.001

The mean serum magnesium levels in the overweight group turned out to be 2.08 ± 0.211 mg/dl and the mean serum magnesium levels in the normal weight group turned out to be 2.55 ± 0.155 mg/dl. The magnesium level in the normal weight group was higher than the overweight and obese group (p-value <0.001). This is shown in Table 2.

**Table 2:** Mean Serum Magnesium Levels in the Study Population

Variable	Overweight and Obese Group	Normal Weight Group	p - value
Serum magnesium level	2.08±0.211	2.55±0.155	<0.001

**Figure 2:**
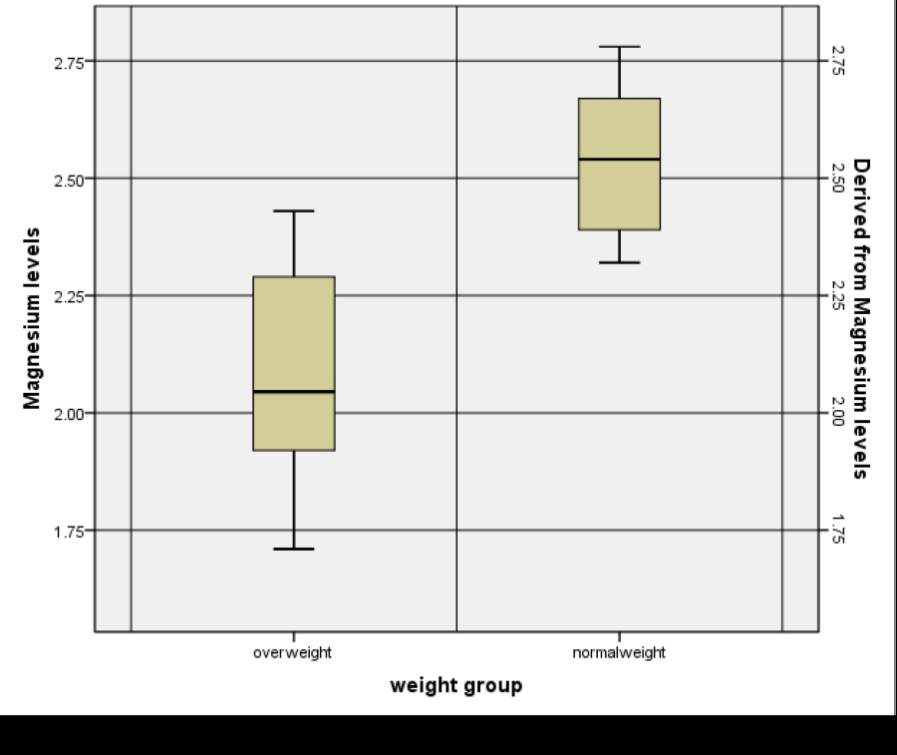
Mean Serum Magnesium Levels in Overweight and Normal Weight Children

The mean serum magnesium levels in different weight groups are represented graphically in Figure 2.

The p value was significant for the mean serum magnesium levels (p-value < 0.001). This shows that the mean serum magnesium levels were lower in the overweight and obese children as compared to normal weight children.

## Discussion

Obesity is a disease with a complex multifactorial pathophysiology. Obesity is spreading like a pandemic worldwide owing to better socioeconomic conditions of the masses, and various researchers have started focusing on the epidemic of obesity in addition to undernutrition. Magnesium has been proposed to be one important micronutrient that plays a key role in regulating the enzymatic processes and glucose metabolism and thus plays a significant role in determining the weight of a person [8]. Overweight individuals, especially adults, have lower serum and intracellular magnesium levels as compared to normal adults. In children, very limited data is present internationally, and in Pakistan, no study has been conducted showing the association of serum magnesium levels with obesity.

Studies done in different health facilities suggest decreased serum magnesium levels as well as a low magnesium-containing diet in obese children. In a study conducted by Huerta, et al., obese children had a lower calorie-adjusted intake of magnesium as compared to normal weight children [9]. Another study conducted by Jose B, et al. [10] showed that overweight children have lower serum magnesium levels (2.12 ± 0.33) as compared to normal weight children (2.56 ± 0.24).

Our findings are consistent with previous studies where it was identified that obese children have an increased incidence of micronutrient deficiencies including magnesium, selenium, cobalt, potassium, zinc, copper, iron, and chromium. According to a study, a negative correlation was observed between serum magnesium levels and the BMI of healthy adults and children [11]. Many other studies done by various researchers showed decreased serum and intracellular levels of magnesium in obese people [9]. A cohort study conducted by Yakinci [12] showed decreased serum magnesium levels in obese children in addition to other micronutrients including copper and zinc. A study done in 2003 by Takaya J, et al. reports decreased intracellular magnesium inside platelets in obese children as compared to healthy controls [13].

Various studies have been done on the relationship between dietary intake and serum levels of magnesium and their relationship with insulin resistance and type 2 diabetes mellitus in adult men and women [14-15]. Another study shows that low serum levels of magnesium can be seen in obese children, despite a high dietary intake of Mg-rich food [16]. This might be due to decreased absorption of magnesium from the intestine or increased excretion. A higher intake of calcium or fats can interfere with magnesium absorption in the gut. The use of tea is higher in our population due to dietary habits. There has been a rise in the consumption of carbonated beverages that can affect the absorption of important nutrients, one of which is magnesium. These habits also decrease appetite and because of this, the intake of healthier foods is also reduced.

Whether magnesium supplementation plays any role in the prevention or cure of obesity is still debatable. A randomized double-blind placebo-controlled trial conducted by Rodríguez-Moran M on obese individuals showed marked improvements in metabolic profiles and blood pressures of obese individuals after magnesium supplementation [17]. A study done showed that obese adolescents have a poorer mineral status (zinc, magnesium, and calcium) than adolescents of normal weight, which can contribute to insulin resistance [18]. Another study also indicated that chromium supplementation along with magnesium leads to a greater decrease in insulin resistance than either ion used alone [19]. Also, it is important to note that most of the fast foods are poor sources of magnesium, and children eating more fast foods are more prone to obesity as evident by the study conducted in Iran recently [20].

Some studies, on the other hand, hold a different point of view. Guerrero-Rumero found that there is no direct relationship between obesity and hypomagnesemia, rather it is hyperglycemia and poor magnesium intake that causes hypomagnesemia irrespective of obesity [21]. As mentioned previously, overweight or obese children are much more likely to remain obese into adulthood. This carries with it an increased risk of many diseases, particularly diabetes mellitus and cardiovascular diseases. The fact that magnesium has beneficial cardiovascular effects makes its supplementation even more important especially in overweight and obese patients as they are at an increased risk of cardiovascular morbidity and mortality.

A randomized, double-blind, placebo-controlled intervention study found that increasing the magnesium intake in overweight and obese adults reduces the arterial stiffness after 24 weeks of supplementation, thus improving the cardiovascular health [22]. Another study reports lower serum magnesium levels in people with existing cardiovascular disease, hypertension, and diabetes as compared to those who were disease free. In participants with no cardiovascular disease, an inverse relationship was noted between magnesium and fasting serum insulin, glucose, systolic blood pressure, and smoking [23]. 

Despite the studies that clearly show a link between serum magnesium levels and obesity, further studies need to be conducted in order to clarify the exact role of magnesium and as to whether the obesity is linked to a decreased intake of magnesium or if the low magnesium level is a direct consequence of obesity. Supplementation of the diet with a micronutrient like magnesium in the prevention of obesity-related complications should also be studied.

## Conclusions

We conclude that overweight and obese children have reduced serum magnesium levels as compared to normal weight children. We speculate that increased weight predisposes the children to magnesium-deficient states and further leads to insulin resistance, which in turn leads to diabetes and increased cardiovascular risks in adulthood. Further studies are required to evaluate the exact role of magnesium intake in the prevention of obesity and to determine a causal relationship between obesity and magnesium deficiency.
